# Quantification of Epithelial Cell Differentiation in Mammary Glands and Carcinomas from DMBA- and MNU-Exposed Rats

**DOI:** 10.1371/journal.pone.0026145

**Published:** 2011-10-12

**Authors:** Deepak Sharma, Bart M. G. Smits, Mark R. Eichelberg, Amanda L. Meilahn, Matthew J. Muelbl, Jill D. Haag, Michael N. Gould

**Affiliations:** McArdle Laboratory for Cancer Research, Department of Oncology, University of Wisconsin School of Medicine and Public Health, University of Wisconsin-Madison, Madison, Wisconsin, United States of America; Baylor College of Medicine, United States of America

## Abstract

Rat mammary carcinogenesis models have been used extensively to study breast cancer initiation, progression, prevention, and intervention. Nevertheless, quantitative molecular data on epithelial cell differentiation in mammary glands of untreated and carcinogen-exposed rats is limited. Here, we describe the characterization of rat mammary epithelial cells (RMECs) by multicolor flow cytometry using antibodies against cell surface proteins CD24, CD29, CD31, CD45, CD49f, CD61, Peanut Lectin, and Thy-1, intracellular proteins CK14, CK19, and FAK, along with phalloidin and Hoechst staining. We identified the luminal and basal/myoepithelial populations and actively dividing RMECs. In inbred rats susceptible to mammary carcinoma development, we quantified the changes in differentiation of the RMEC populations at 1, 2, and 4 weeks after exposure to mammary carcinogens DMBA and MNU. DMBA exposure did not alter the percentage of basal or luminal cells, but upregulated CD49f (Integrin α6) expression and increased cell cycle activity. MNU exposure resulted in a temporary disruption of the luminal/basal ratio and no CD49f upregulation. When comparing DMBA- or MNU-induced mammary carcinomas, the RMEC differentiation profiles are indistinguishable. The carcinomas compared with mammary glands from untreated rats, showed upregulation of CD29 (Integrin β1) and CD49f expression, increased FAK (focal adhesion kinase) activation especially in the CD29hi population, and decreased CD61 (Integrin β3) expression. This study provides quantitative insight into the protein expression phenotypes underlying RMEC differentiation. The results highlight distinct RMEC differentiation etiologies of DMBA and MNU exposure, while the resulting carcinomas have similar RMEC differentiation profiles. The methodology and data will enhance rat mammary carcinogenesis models in the study of the role of epithelial cell differentiation in breast cancer.

## Introduction

The rat is a well-established model organism to study breast cancer etiology, prevention and treatment. The chemical carcinogens 7,12-dimethylbenz(a)anthracene (DMBA) or *N*-methyl-*N*-nitrosourea (MNU) were recognized in the 1960 s and 1970 s, respectively, to be capable of inducing rat mammary cancer [1], [2] and have become the most commonly used mammary carcinogens. In susceptible rat strains, such as Wistar-Furth (WF) and Spraque-Dawley (SD), a single dose of carcinogen is capable of rapidly inducing multiple mammary carcinomas. Rat mammary carcinomas display many features of human adenocarcinoma of the breast, such as histological progression [3], [4] and ovarian hormone dependence [5], [6]. In the DMBA-induced rat mammary carcinogenesis model, Russo and Russo identified the terminal end bud (TEB) structure in the mammary epithelium to be associated with increased proliferation along with the formation of intraductal proliferation, in-situ carcinomas, and invasive carcinomas [7]. MNU carcinogenesis has been found to be capable of generating invasive carcinomas as well as early stage mammary lesions [8]. In our laboratory DMBA- and MNU-induced rat mammary carcinogenesis models are used to decipher the genetic basis of breast cancer susceptibility. We have identified multiple loci modulating mammary carcinoma multiplicity in rats, of which some have been shown to affect breast cancer risk in women [9]. These highly relevant rat models allow us to explore mechanisms through which these loci modulate breast cancer susceptibility, however, minimal molecular data are currently available on the cellular composition of the rat mammary epithelium and on quantification of the changes of protein expression phenotypes underlying rat mammary epithelial cell (RMEC) differentiation throughout the process of chemical carcinogenesis.

The cellular composition of the mouse mammary epithelium has been characterized in detail by multicolor flow cytometry using antibody staining of cell surface and internal proteins on gently digested, monodispersed mammary glands. The epithelial hierarchy within the mammary gland is thought to start with an undifferentiated mammary stem cell (MaSC) that maintains itself through self-renewal and is capable of differentiating into committed progenitors [10], [11]. These progenitors ultimately give rise to progeny that are the mature ductal and alveolar cells, which belong to the luminal epithelial cell lineage lining the lumen of the mammary gland, and the basal cells surrounding the luminal epithelium and contacting the basement membrane (reviewed in: [12]). Luminal cells have secretory properties. In the mouse mammary gland, luminal cells have been shown to express heat stable antigen (HSA; CD24) and intermediate (med) levels of Integrin β1 (CD29) and cytokeratin (CK) 19, but not CK14 (CD24+CD29med, CK19+CK14-) [11], [13]. Within the luminal population, luminal progenitor cells have been described to express Integrin β3 (CD61) [14]. Basal cells include mature myoepithelial cells and have contractile muscle as well as epithelial properties. In the mouse mammary gland, these cells are typically identified by the expression of CD24, high (hi) levels of CD29, CK14, smooth muscle actin (SMA) but not CK19 (CD24+CD29hi, CK14+CK19-) [11], [13], [15]. Although a unique molecular marker for the MaSC has not been identified, single mammary epithelial cells capable of repopulating a mammary-free fat pad have been shown to be enriched in a CD24+CD29hi population of cells expressing high levels of Integrin α6 (CD49f), and lack expression of stem cell antigen (SCA) 1 [10], [11].

In various transgenic mouse models, a role for aberrant mammary epithelial cell differentiation in mammary carcinogenesis was recognized. For example, in preneoplastic mammary glands of transgenic mice expressing the *wnt-1* oncogene under control of the Mouse Mammary Tumor Virus (MMTV) promoter, the percentage of mammary epithelial cells highly expressing CD29 is increased [11]. Earlier, it was shown that ablation of Integrin β1 abolished mouse mammary tumor development [16]. Integrin β1 has been shown to affect proliferation and differentiation in the luminal lineage [17] and to be essential for MaSC repopulation ability [18]. Similarly, targeted ablation in the mammary epithelium of focal adhesion kinase (FAK), a cytoplasmic tyrosine kinase and important mediator of Integrin signaling, significantly suppresses mammary carcinoma incidence in the mouse MMTV-PyVT model by affecting the pool of MaSC in the untransformed mammary gland and mammary cancer stem cells (MaCSC) in the primary tumors [19], [20]. FAK is known to affect many cellular processes, including survival, proliferation, and differentiation (reviewed in [21]).

In this study, we used multicolor flow cytometry to annotate the luminal and basal/myoepithelial populations of RMECs. We quantified the protein expression phenotypes underlying these populations in mammary glands isolated at 1, 2, and 4 weeks after DMBA or MNU exposure as well as in carcinomas and mammary glands from untreated age-matched control animals of a highly susceptible congenic recombinant inbred rat line. Following exposure of the rats to the mammary carcinogens DMBA or MNU, the RMECs showed a distinct cellular differentiation etiology, while the carcinomas resulting from DMBA- or MNU-induced carcinogenesis have a very similar cellular differentiation profile.

## Results

### Characterization of RMEC populations

**Figure 1:**
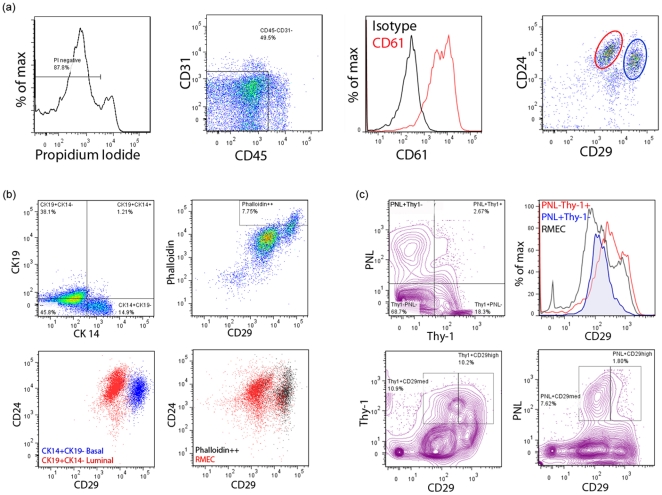
Characterization of rat mammary epithelial cells (RMECs) based on cell surface and intracellular markers. (**A**) Representative flow cytometric histograms and dot plots showing gating for propidium iodide (PI)-negative (live) cells (left panel); exclusion of endothelial cells and leukocytes based on CD31 and CD45 expression, respectively (middle left panel); CD61 expression in CD45–CD31– RMECs (middle right panel); CD24 and CD29 expression in CD45–CD31– RMECs identifies two major populations indicated with a red or blue circle (right panel). (**B**) Dot plots of intracellular cytokeratin (CK) 14 and CK19 expression in CD45–CD31– RMECs (upper left panel); intracellular smooth muscle actin (SMA) staining with phalloidin and CD29 expression in CD45–CD31– RMECs (upper right panel); overlay of dot plots showing CD24 and CD29 expression in CK14+CK19- cells and CK19+CK14- cells (lower left panel); overlay of dot plots of phalloidin bright cells on CD24 and CD29 expression in CD45–CD31– RMECs (lower right panel). Based on CK14, CK19, and SMA expression, the luminal (red) and basal (blue) populations in CD45–CD31– RMECs are identified. (**C**) Contour plot showing binding of Peanut Lectin (PNL) or anti-Thy-1 in CD45–CD31– RMECs (left panel), overlaid histograms showing CD29 expression on PNL+Thy1-, PNL-Thy-1+ cells (middle left panel), contour plots showing anti-Thy-1 (middle right panel) or PNL binding in CD29med or CD29hi cells (right panel). For all panels, rats of 12 weeks of age were used.

We optimized a protocol to obtain single cells from rat mammary glands and frank mammary carcinomas. After the gentle digestion procedure, each mammary gland sample yielded approximately 8 million mononucleated cells that were aliquoted for antibody staining and multicolor flow cytometric analysis. In the analysis of the flow cytometric profiles, single cells were discriminated from sticking cells based on forward scatter and side scatter width. The live cells were gated using propidium iodide dye exclusion (PI-negative; Fig. 1A). The rat mammary epithelial cells (RMECs) were separated from hematopoietic and endothelial cells based on lack of CD45 and CD31 expression, respectively (Fig. 1A). The majority (71.4±8.2%) of CD45-CD31- cells expressed CD61 (Fig. 1A), but CD61 expression does not segregate a population. Based on expression of CD24 and CD29, the RMECs could be divided into two distinct major populations which showed CD24+CD29hi or CD24+CD29med phenotypes (Fig. 1A). Intracellular staining with CK14 and CK19 identified basal cells (CK14+CK19-) in the CD24+CD29hi population and luminal cells (CK19+CK14-) in the CD24+CD29med population (Fig. 1B). SMA expression as evinced from phalloidin staining identified myoepithelial cells the in CD24+CD29hi population (Fig. 1B). Based on these parameters, CD24+CD29med cells were identified as luminal and CD24+CD29hi cells as basal (including myoepithelial) RMECs.

In the past, RMEC populations with differential clonogenic capabilities were identified using PNL and anti-Thy-1 staining [22]. In our experiments, PNL and anti-Thy-1 yielded good separation of RMEC populations (Fig. 1C). Approximately 10% of the RMECs was found to be PNL+Thy-1-, approximately 18% was PNL-Thy-1+, approximately 3% was PNL+Thy-1+, and the vast majority of the RMECs did not stain with PNL or anti-Thy-1 (Fig. 1C). The PNL+ population was found to overlap with the CD29med population, while the Thy-1+ population overlapped equally between the CD29med and CD29hi populations (Fig. 1C).

### CD49f expression is upregulated in actively dividing cells

**Figure 2:**
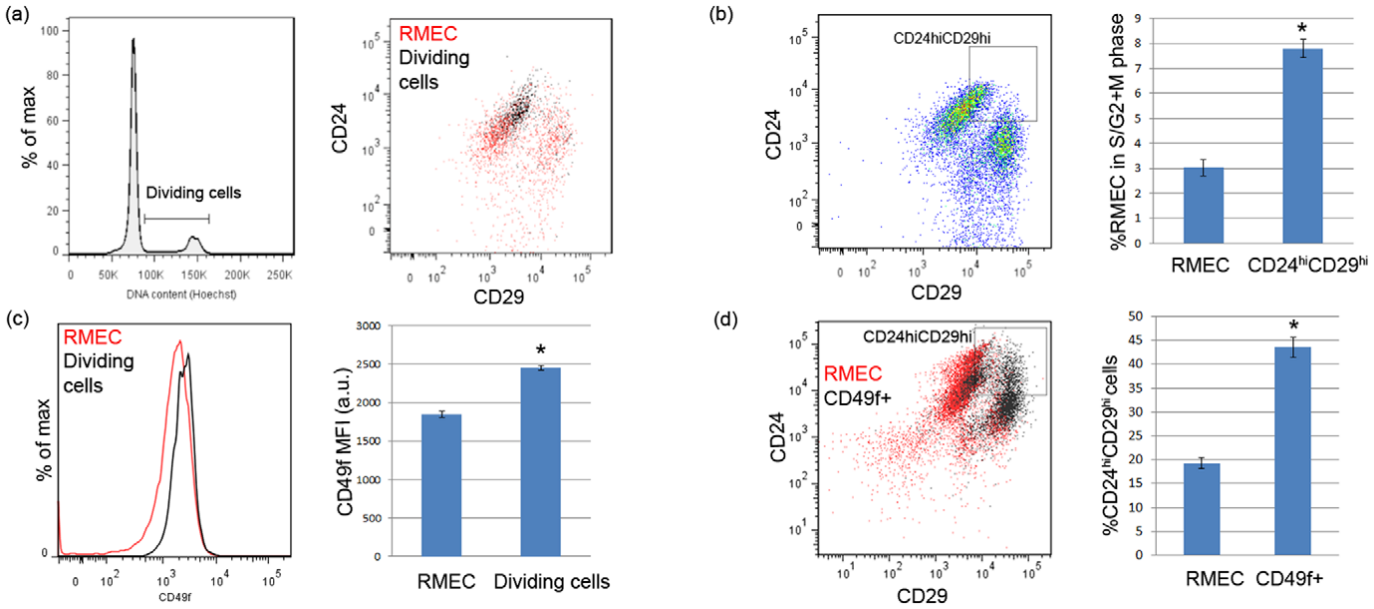
Features of the actively dividing cells in CD45–CD31– RMECs. (**A**) Flow cytometric histogram showing gating for actively dividing cells (cells in S/G2+M phase of the cell cycle) by having >2n cellular DNA content (left panel); representative dot plot showing the actively dividing cells overlaid on CD24 and CD29 expression in the RMECs (right panel). (**B**) Representative dot plot showing gating for RMECs expressing high levels of both CD24 and CD29 (CD24hiCD29hi gate; left panel); bar graph (right panel) showing mean ± sem percentage of cells in S/G2+M phase of the cell cycle in total RMECs or CD24hiCD29hi-gated cells (n = 24 each). A significant enrichment of actively dividing cells was detected in the CD24hiCD29hi-gated cells (p<0.05; indicated with an asterisk). (**C**) Overlaid histogram showing CD49f expression in the total RMECs and actively dividing cells (left panel); bar graph (right panel) showing mean fluorescence intensity (MFI) in artificial units (a.u.) ± sem of CD49f in the total RMECs and actively dividing cells (n = 14 each). Significantly different MFI is indicated with an asterisk (p<0.05). (**D**) Representative dot plot showing CD49f expressing cells (CD49f+) overlaid on CD24 and CD29 expression of the RMECs (left panel); bar graph showing mean ± sem percentage of CD24hiCD29hi-gated cells in total RMECs or the CD49f expressing population (right panel). A significant enrichment of CD24hiCD29hi-gated cells was detected in the CD49f expressing population (p<0.05; indicated with an asterisk). For all panels, rats of 12 weeks of age were used.

We evaluated the location of actively dividing RMECs on the luminal/basal (as defined by CD24 and CD29 expression) flow cytometric profile. Actively dividing cells (in S/G2+M phase of the cell cycle) were identified based on more than 2n cellular DNA content using Hoechst staining (Fig. 2A). These cells appear to be located in the ‘upper right‘ quadrant of the luminal/basal profile, expressing highest levels of CD24 and CD29 (Fig. 2A). Using a gating strategy to quantify this observation, the CD24hiCD29hi gate was strongly enriched (∼300%) in actively dividing cells, as compared with total RMECs (Fig. 2B). Besides expressing high levels of CD24 and CD29, the actively dividing cells also expressed elevated CD49f levels as compared with total RMECs (Fig. 2C). Accordingly, CD49f+ cells were found to contain a higher percentage of CD24hiCD29hi-gated cells as compared with total RMECs (Fig. 2D).

### RMEC differentiation and proliferation following DMBA or MNU treatment

**Figure 3:**
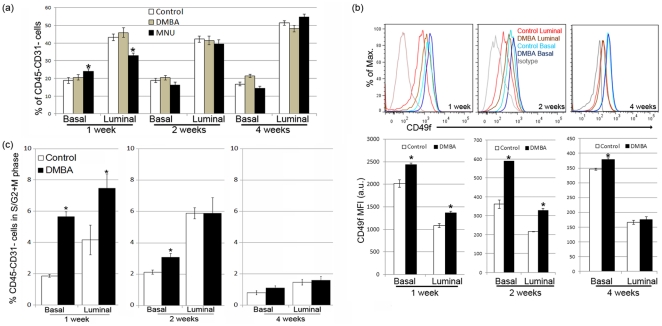
Modulation of RMEC differentiation by exposure of the rats to 7,12-dimethylbenz(a)anthracene (DMBA) or *N*-methyl-*N*-nitrosourea (MNU). (**A**) Mean ± sem percentage of basal and luminal RMEC populations in age-matched untreated control, DMBA-treated, or MNU-treated rats at 1, 2, and 4 weeks after treatment. 1 week: control n = 21, DMBA n = 7, MNU n = 12; 2 weeks: control n = 18, DMBA n = 9, MNU n = 10; 4 weeks: control n = 24, DMBA n = 10, MNU n = 12. A significantly different percentage as compared to the age-matched control group is indicated with an asterisk (p<0.05). (**B**) Representative overlaid histograms (upper panels) showing upregulation of CD49f expression in basal and luminal cells following DMBA treatment; bar graphs (lower panels) quantifying mean fluoresence intensity (MFI) in artificial units (a.u.) ± sem of CD49f in basal and luminal RMECs from rats 1, 2 or 4 weeks after DMBA treatment as compared to age-matched untreated control rats. 1 week: control n = 6, DMBA n = 6; 2 weeks: control n = 6, DMBA n = 6; 4 weeks: control n = 9, DMBA n = 7. Significantly different MFI comparing the DMBA group to the control group is indicated with an asterisk (p<0.05). (**C**) Bar graphs showing the mean ± sem percentage of RMECs containing >2n cellular DNA (dividing cells in S/G2+M phase of cell cycle) in age-matched untreated control or DMBA-treated rats 1, 2, and 4 weeks after treatment. A significantly different percentage comparing the DMBA group to the control group is indicated with an asterisk (p<0.05).

Following the characterization of the RMECs in untreated control rats, we treated female rats with the mammary carcinogens DMBA or MNU and quantified the protein expression phenotypes underlying the RMEC populations at 1, 2, or 4 weeks after treatment. Administration of DMBA to the rats did not significantly alter the percentage of luminal and basal cells as compared with the untreated control rats at any time point measured (Fig. 3A). On the other hand, MNU administration significantly altered the percentages of basal and luminal cells at 1 week after treatment (Fig. 3A). At 2 and 4 weeks after treatment, the percentages of basal and luminal cells from the MNU-treated rats were at similar levels compared to those from the untreated control rats (Fig. 3A).

The expression of CD49f was upregulated on basal as well as luminal cells 1 and 2 weeks after DMBA treatment (Fig. 3B). CD49f expression remained high on the basal cells from DMBA-treated animals 4 weeks after treatment, but in luminal cells CD49f expression returned to similar levels compared to untreated control rats at this same time point (Fig. 3B). MNU treatment had no effect on CD49f expression in the RMECs at any time point studied (data not shown).

Along with the CD49f upregulation, a significantly higher percentage of actively dividing cells was seen in luminal as well as basal RMECs from DMBA-treated rats as compared to untreated control rats at 1 week after treatment (Fig. 3C). At 2 weeks after DMBA treatment, only the basal but not the luminal cells showed a significantly higher percentage of actively dividing cells in the DMBA-treated rats as compared with the untreated control rats (Fig. 3C). At 4 weeks of age, there was no difference in the percentage of actively dividing cells between the (luminal as well as basal) RMECs from DMBA-treated rats compared to untreated control rats (Fig. 3C). At all time points, there was a higher percentage of luminal cells in the S/G2+M phase of the cell cycle as compared to basal cells (Fig. 3C).

### RMEC differentation in chemically induced mammary carcinomas

**Figure 4:**
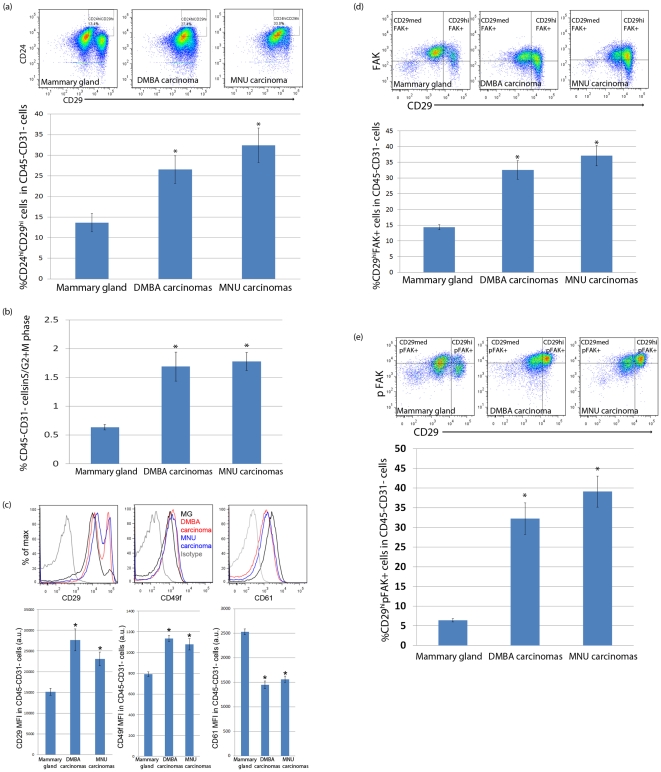
Differences between the RMECs from mammary glands of untreated control rats and mammary carcinomas from rats exposed to 7,12-dimethylbenz(a)anthracene (DMBA) or *N*-methyl-*N*-nitrosourea (MNU). (**A**) Representative pseudo-color dot plots showing CD24 and CD29 expression in the RMECs from the mammary gland of an age-matched (22 weeks of age) untreated control rat (upper left panel) and a DMBA- (upper middle panel) or MNU-induced (upper right panel) carcinoma; bar graphs (lower panel) quantifying mean ± sem percentage cells in the CD24hiD29hi gate within the total (CD45–CD31–) RMECs. A significantly different percentage comparing carcinomas to mammary glands is indicated with an asterisk (p<0.05). (**B**) Bar graphs showing the mean ± sem percentage of RMECs containing >2n cellular DNA (actively dividing cells in S/G2+M phase of cell cycle). Significantly different percentage comparing RMECs from carcinomas to control mammary glands is indicated with an asterisk (p<0.05). (**C**) Representative overlaid histograms showing upregulation of CD29 expression (upper left panel), upregulation of CD49f expression (upper middle panel) and downregulation of CD61 expression (upper right panel) in RMECs of a DMBA-induced or MNU-induced carcinoma as compared to a control mammary gland; bar graphs quantifying the mean fluorescence intensity (MFI) in artificial units (a.u.) ± sem of CD29 (lower left panel), CD49f (lower middle panel) and CD61 (lower right panel) on RMECs from control mammary glands and carcinomas. Significantly different MFI is indicated with an asterisk (p<0.05). (**D**) Representative pseudo-color dot plot showing gating for CD29 and focal adhesion kinase (FAK) in RMECs from a control mammary gland (upper left panel), a DMBA-induced (upper middle panel) and MNU-induced (upper right panel) carcinoma; bar graph (lower panel) quantifying mean ± sem percentage of CD29hiFAK+ cells. A significantly different percentage comparing carcinomas to control mammary glands is indicated with an asterisk (p<0.05). (**E**) Representative pseudo-color dot plot showing gating for CD29 and Y397-phosphorylated focal adhesion kinase (pFAK) in RMECs from a control mammary gland (upper left panel), a DMBA-induced (upper middle panel) and MNU-induced (upper right panel) carcinoma; bar graph (lower panel) quantifying mean ± sem percentage of CD29hi pFAK+ cells. A significantly different percentage comparing carcinomas to control mammary glands is indicated with an asterisk (p<0.05). In the entire figure 4, age-matched untreated control mammary glands: n = 16, DMBA-induced mammary carcinomas: n = 10 and MNU-induced mammary carcinomas: n = 10.

We also evaluated the protein expression profile of the RMECs from carcinomas and mammary glands from untreated control rats of the same age (15 weeks after carcinogen exposure). The RMECs from DMBA- or MNU-induced carcinomas appear to be highly similar to each other in regard to their cellular differentiation profile, but were different from the RMECs from the mammary gland of the untreated control animals (Fig. 4A). The RMECs from carcinomas have higher percentages of cells in the CD24hiCD29hi gate as compared to the RMECs from the mammary gland of the control rats (Fig. 4A). The percentage of actively dividing cells was also increased in the RMECs from carcinomas as compared to the RMECs from the mammary gland of the control rats (Fig. 4B). In addition, the RMECs from carcinomas showed increased expression of CD29 and CD49f, along with a decreased expression of CD61 (Fig. 4C). Finally, the percentage of myoepithelial cells that stained brightly with phalloidin, as well as Thy-1 expression were decreased in the RMECs from carcinomas as compared to those of the mammary gland of untreated control rats (data not shown).

Focal adhesion kinase (FAK) has been shown to signal downstream of Integrins [23], [24]. As Integrins α6 (CD49f) and β1 (CD29) were upregulated and Integrin β3 (CD61) was downregulated in the RMECs from carcinomas, we checked if FAK and Y397-phosphorylated FAK (pFAK) levels were affected in the RMECs from carcinomas. In the mammary gland from untreated control rats, the majority of the RMECs expressed intracellular FAK (Fig. 4D) and a smaller portion of the cells expressed the phosphorylated form of FAK (Fig. 4E). Both FAK and pFAK levels appear higher in the CD29med population as compared with the CD29hi population. The expression of FAK was specifically upregulated in CD29hi RMECs from carcinomas as compared to CD29hi RMECs from the mammary gland of untreated control rats (Fig. 4D), which was even more pronounced for pFAK (Fig. 4E).

## Discussion

Rat chemical carcinogenesis models for breast cancer have been used extensively in preclinical research. The human breast and rat mammary gland have a similar ductal-lobular organization and mammary cancers induced in the rat are predominantly hormone-dependent and of ductal origin, similar to the majority of human breast cancers [3], [7]. The two most widely used mammary carcinogens are the polycyclic hydrocarbon 7,12-dimethylbenz(a)anthracene (DMBA) and the directly alkylating agent *N*-methyl-*N*-nitrosourea (MNU). DMBA, unlike MNU, requires metabolic activation to become mutagenic [25]. In this study, we first characterized the cellular differentiation profile of the rat mammary epithelial cells (RMECs). Subsequently, we focused on quantifying the changes of RMEC differentiation in the mammary gland following exposure to DMBA or MNU and in the resulting carcinomas as compared with mammary glands from untreated control animals.

Multicolor flow cytometric profiles of mammary epithelial cells (MECs) have been described extensively in the mouse, but not in the rat. In our study, RMECs showed features similar to those of mice including cell surface expression of CD24 and CD29, defining the luminal and basal populations [11]. The luminal cells showed a CD24+CD29med phenotype and expressed intracellular CK19. Basal cells, the other dominant population in MECs, showed a CD24+CD29hi phenotype and expressed intracellular CK14. A subset of the basal cells, the myoepithelial cells, showed bright staining with phalloidin indicating smooth muscle actin (SMA) expression. A clear difference between the rat and mouse profiles is that the rat basal population (with respect to the rat luminal population) appears to express higher levels of CD29 as compared to the mouse basal population (with respect to the mouse luminal population). Subsequently, a distinct population enriched in mammary stem/progenitor cells that has been shown to express higher CD29 levels than the basal cell population in mice [11], could not be identified in the rat. Another difference between the mouse and rat MEC characterizations is the expression pattern of CD49f and CD61. In the mouse high expression of CD49f has been shown to define the basal population and, together with high expression of CD24, define a MaSC-enriched population [10]. CD61 expressing cells in the mouse mammary gland, together with low expression of CD29, define the luminal progenitor pool [14]. In the rat, expression of both CD49f and CD61 are detectable, but do not separate populations of RMECs, again underscoring interspecies differences in the protein expression profile of the MECs.

We followed a previously published study in the rat that used PNL and anti-Thy-1 staining to fractionate the RMECs [22]. That study identified the PNL+ population to be enriched in clonogenic cells, i.e. cells capable of forming alveolar units after transplantation. PNL has also been shown to stain most alveolar epithelial cells and luminal alveolar cells [26], [27]. Here, we verify that the PNL+ and Thy-1+ populations are segregating populations of RMECs. The PNL+ population was found highly enriched in the CD29med population as compared to the CD29hi population, indicating that the clonogenic PNL+ population coincides with the luminal cells, perhaps defining a pool of alveolar progenitor cells, which has been suggested to underlie clonogenicity of a small fraction of the luminal cells in mice [12], [14]. In contrast to the PNL+ population, we found the non-clonogenic Thy-1+ population to equally overlay the CD29med and CD29hi populations. Thy-1 has previously been found to be present on and immediately adjacent to the myoepithelial cells of the ducts and alveoli [28]. As we found SMA expression (a marker for myoepithelial cells) exclusively in CD29hi cells of the basal RMEC population, we hypothesize that either a myoepithelial cell population defined by Thy-1 can also be found in the luminal population, or Thy-1+ cells define a population other than myoepithelial cells, such as cells of mesenchymal origin as suggested earlier [22], [28].

In this study, we find the luminal population to have a higher percentage of cells in S/G2+M phase of the cell cycle as compared with the basal population. These actively dividing cells appeared to be enriched in cells expressing high levels of both CD24 and CD29 (CD24hiCD29hi-gated cells), as compared with the total RMECs. Interestingly, these CD24hiCD29hi-gated cells also express elevated levels of CD49f, a marker previously associated with the MaSC population in mice of which the majority of cells appeared to be cycling [10]. These findings suggest that a putative rat MaSC-like population may be located in the luminal population, likely consisting of those luminal cells expressing the highest levels of CD29 and CD49f.

**Figure 5:**
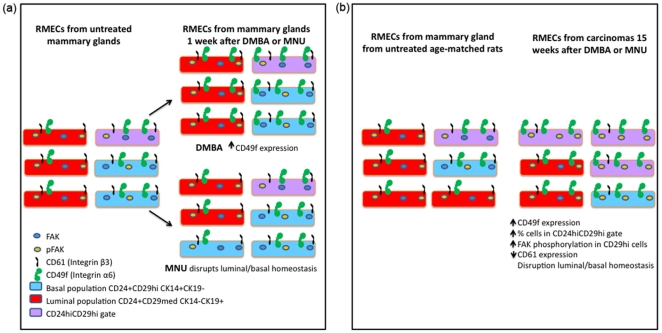
Epithelial cell differentiation in mammary glands and carcinomas from 7,12-dimethylbenz(a)anthracene (DMBA)-or *N*-methyl-*N*-nitrosourea (MNU)-exposed rats. (**A**) Schematic representation of the modulation of RMEC differentiation 1 week after exposure of rats to the mammary carcinogens DMBA or MNU. DMBA exposure increases CD49f expression and proliferation (not shown here). MNU exposure disrupts the luminal and basal homeostasis. (**B**) Schematic representation of the changes of RMEC differentiation in carcinomas as compared to mammary gland from untreated age-matched (22 weeks of age) control rats. Note that the RMECs from animals of 22 weeks of age have a higher percentage of luminal cells as compared to younger animals (comparing Fig. 1A to Fig. 4A). In both panels cell surface expression of CD24, CD29, CD49f and CD61 and intracellular expression of focal adhesion kinase (FAK) and Y397-phosphorylated FAK (pFAK) in basal, luminal and CD24hiCD29hi-gated cells are shown.

The focus of this study was to quantify changes in the differentiation of the RMECs 1, 2, and 4 weeks after exposure to DMBA and MNU and changes in RMEC differentiation comparing carcinomas to mammary gland from untreated control rats. DMBA treatment did not modify the percentages of RMECs in the luminal or basal populations, but MNU treatment drastically disrupted the luminal and basal cell populations within 1 week (Fig. 5A). The MNU-induced increase in basal cells and decrease in luminal cells was possibly corrected by homeostatic mechanisms as these populations returned to levels equal to those of untreated control rats within four weeks after exposure. On the other hand, DMBA treatment resulted in significant upregulation of CD49f, which was still detectable in the basal population 4 weeks after exposure. These observations underscore the differential etiology of mammary carcinogenesis induced by DMBA and MNU exposure. We hypothesize that DMBA, besides having a mutagenic effect after metabolic activation, drives mammary carcinogenesis by increasing RMEC proliferation and modulating CD49f-related differentiation. This hypothesis is supported by a previous observation of expansion of terminal end bud structures in mammary glands of DMBA-treated rats [3], which are the structures associated with high rates of proliferation [29]. A previous gene expression analysis on mammary glands of DMBA-treated rats also supports this hypothesis, as a number of cell cycle related genes, such as *PCNA*, *Igf1*, *Igfbp2*, and *Ran* were found to be upregulated as compared with mammary glands of untreated rats [30]. MNU is likely acutely toxic to the luminal cells, which could explain the immediate disruption of RMEC luminal/basal homeostasis. We have previously shown killing of RMEC clonogens directly after MNU exposure [31] and localization of the clonogen-enriched PNL+ cell population in the luminal population (this study), making this hypothesis plausible.

Interestingly, despite the etiological distinction of RMEC differentiation between exposure to the carcinogens DMBA and MNU, the RMEC protein expression profiles of the mammary carcinomas induced by the two carcinogens look similar. It should be noted that on the molecular genetic level several differences between DMBA- and MNU-induced mammary carcinomas have been reported, including subtle carcinogen-specific expression signatures irrespective of the histological grade of the carcinomas [32], and frequent occurrence of Ha-Ras codon-12 mutations in MNU-induced mammary carcinomas as compared to absence of such mutations in DMBA-induced mammary carcinomas [33].

When comparing the mammary carcinomas to the age-matched untreated control mammary gland, the RMECs from mammary carcinomas showed increased expression of Integrin β1, Integrin α6, FAK, and pFAK (Fig. 5B), as well as decreased expression of Integrin β3 (Fig. 5B), SMA, and Thy-1. We noticed a shift of the RMECs of carcinomas towards the CD24hiCD29hi gate, resembling the CD24/CD29 profile observed in premalignant mammary glands of transgenic MMTV-*Wnt1* mice [11]. In addition, the RMECs from mammary carcinomas have a higher percentage of cells in S/G2+M phase of the cell cycle. As the RMECs from mammary carcinomas also express higher levels of Integrin α6 (CD49f), which is a marker previously associated with MaSCs [10], show increased proliferation, and show increased pFAK expression that was also found be associated with a stem-progenitor cell pool [20], we hypothesize that DMBA- and MNU-induced mammary carcinomas may have an increased pool of stem/progenitor-like cells driving tumorigenesis. In the future, the existance of such a population, as well as its tumorigenic potential would have to be verified in transplantation studies.

Mammary specific ablation of FAK was earlier reported to ameliorate mammary tumor progression in mice [34] by affecting the mammary cancer stem/progenitor cells [20]. Early work has demonstrated that FAK autophosphorylation at Y397 is strongly increased upon interaction with activator proteins, such as Integrin βs, rendering pFAK in its active state (recently reviewed in [21]). We found in the mammary gland of untreated control rats that about 60% of the RMECs stained positive for intracellular FAK and about 35% of RMECs stained positive for pFAK. DMBA- and MNU-induced mammary carcinomas showed specific upregulation of FAK expression and FAK autophosphorylation in CD29hi (basal) cells. The upregulation of Integrin β1 and α6, as well as the activation of FAK in the mammary carcinomas may provide an important research tool for potential use of rat carcinogenesis models for preclinical evaluation FAK- and/or Integrin-signaling inhibitors as anticancer drugs.

The results provide detailed insights into different populations of RMECs. Importantly, the methodology established in this study allowed us to quantify changes in RMEC differentiation in the process of chemical carcinogenesis with the two most commonly used mammary carcinogens, namely DMBA and MNU. We also noticed an effect of age on the RMEC differentiation profile, as rats of 22 weeks of age (Fig. 4A) have a higher percentage of luminal cells as compared with rats of 12 weeks of age (Fig. 1A; also schematically presentated in Fig. 5B). In the future, the contribution of age to RMEC differentiation would have to be addressed in a separate study. Detailed knowledge of changes in RMEC differentiation after carcinogen exposure is important to understand the cellular differentiation states associated with genetic and/or environmentally-induced susceptibility to breast cancer for which the rat is a widely studied model organism.

## Materials and Methods

### Animals

All animal experiments were conducted at our facility under protocol number M01353–0–07–10 approved by the University of Wisconsin Medical School Animal Care and Use Committee. All studies described here were conducted using susceptible congenic recombinant inbred rat line (WF.WKy) derived from a congenic line (Line O) that was originally developed on the Wistar-Furth (WF) genetic background carrying selected Wistar-Kyoto (WKy) alleles [9]. This susceptible congenic control line, WF.WKy, harbors the WF genotype across all *Mcs* loci and displays the same mammary carcinoma multiplicity phenotype as the susceptible inbred WF strain [9]. Rats were maintained in a 12-hour light/12-hour dark cycle and were provided with Teklad lab blox chow and acidified water *ad libitum.*

### DMBA/MNU treatment

At 49–57 days of age, WF.WKy female rats were administered 7,12-dimethylbenz(a)anthracene (DMBA; ACROS Organics, Fisher Scientific) in sesame oil as a single gastric intubation of 65 mg/kg of body weight or were injected intraperitonially with *N*-methyl-*N*-nitrosourea (MNU; Sigma) at 50 mg/kg of body weight. DMBA or MNU treatment of the susceptible WF.WKy rats routinely yields ∼8 or ∼4 mammary carcinomas per rat at 15 weeks post-treatment, respectively [35]. The treated and age-matched untreated control rats were sacrificed 1, 2, 4 or 15 weeks after carcinogen exposure and their mammary glands or frank mammary carcinomas were isolated.

### Isolation of mammary epithelial single cells

The protocol used to isolate and monodisperse RMECs was optimized earlier [36]. For each rat, tissue from both abdominal and the adjacent inguinal glands with lymph nodes (LN) excised, or frank mammary carcinomas was kept separately and finely scissor-minced over ice. Each minced mammary gland or mammary carcinoma sample was exposed to 10 ml or 5 ml, respectively, of GIBCO DMEM/F12 (Invitrogen) containing 0.01 g/ml of type III Collagenase (Worthington) for 2 hours at 37°C under gentle horizontal shaking. DNAseI (Worthington) was added to 0.2 µg/ml and the samples were incubated for 10 min under vigorous shaking. Fat was removed from the pelleted cell fraction by pipetting and the pellet was washed once with 10 ml of DMEM/F12. To monodisperse, the cells were pelleted and dissolved in 2 ml prewarmed (37°C) Hanks' Balanced Salt Solution (HBSS, GIBCO) containing 0.025% (w/v) of Trypsin (Worthington), and 6.8 mM of EDTA. After 5 min of trypsin treatment, 4 ml of DMEM/F12 containing 10% of Fetal Bovine Serum (FBS; Hyclone) was added to stop the reaction. The cells were spun, resuspended in 2 ml of DMEM/F12 containing 10% FBS and passed through a 40 µm cell strainer. To rinse the centrifugation tube, another 2 ml of DMEM/F12 with 10% FBS was added and passed through the same cell strainer.

### Antibody staining of RMEC

To stain the single cells, rat antibodies against CD49f (Santa Cruz), CD24, CD29, CD31, CD45, CD61, and Thy-1 (BD pharmingen) or Peanut Lectin (Sigma) were used. Antibodies against CD24, CD31 and CD49f were not available for the rat with the desired fluorochrome so these were conjugated in our laboratory with amine reactive DyLight dyes (with excitation wavelength 405 nm, 550 nm, 633 nm or 680 nm) using DyLight Microscale labeling kits following manufacturer's instructions (Pierce). Freshly isolated single cells were incubated with antibodies for 20 min on ice for surface staining, washed and fixed in 1% paraformaldehyde. The stained samples were acquired on a BD LSR II flow cytometer equipped with 4 lasers (multi-line UV, 405 nm, 488 nm and 633 nm). A subset of stained and fixed cells were permeabilized with 0.5% Tween-20 (15 min, at room temp) followed by 0.5% Triton-X100 (5 min, at room temp) and were stained with fluorochrome conjugated antibodies against rat CK14 and CK19 (Abcam) or FAK (Cell Signaling Technologies) and Alexa Fluor-532-phalloidin (Invitrogen). Cells were stained with Hoechst33342 (10 µg/ml) for 2 hr. Hoechst fluorescence (350 nm excitation/450 nm emission, linear scale) was used for cellular DNA content and cell cycle analysis. Propidium iodide was added immediately before acquiring the samples. The cells were acquired on a BD FACS Aria flow cytometer equipped with 5 lasers (multi-line UV, 405 nm, 488 nm, 540 nm and 640 nm). The data were collected as fcs3 files using FACS Diva software and analyzed using FlowJo software (Treestar Inc). Data files obtained from cell samples stained with single antibodies and control unstained cell samples were used for compensation. Data on percentages of cells in various gated populations or Mean Fluorescence Intensities (MFI) of entire populations were exported and statistically analyzed using Student's t-test in MS Excel.

## References

[1] Huggins C, Grand LC, Brillantes FP (1961). Mammary cancer induced by a single feeding of polymucular hydrocarbons, and its suppression.. Nature.

[2] Gullino PM, Pettigrew HM, Grantham FH (1975). N-nitrosomethylurea as mammary gland carcinogen in rats.. J Natl Cancer Inst.

[3] Russo J, Gusterson BA, Rogers AE, Russo IH, Wellings SR (1990). Comparative study of human and rat mammary tumorigenesis.. Lab Invest.

[4] Singh M, McGinley JN, Thompson HJ (2000). A comparison of the histopathology of premalignant and malignant mammary gland lesions induced in sexually immature rats with those occurring in the human.. Lab Invest.

[5] Nandi S, Guzman RC, Yang J (1995). Hormones and mammary carcinogenesis in mice, rats, and humans: a unifying hypothesis.. Proc Natl Acad Sci U S A.

[6] Thompson HJ, McGinley J, Rothhammer K, Singh M (1998). Ovarian hormone dependence of pre-malignant and malignant mammary gland lesions induced in pre-pubertal rats by 1-methyl-1-nitrosourea.. Carcinogenesis.

[7] Russo J, Russo IH (1996). Experimentally induced mammary tumors in rats.. Breast Cancer Res Treat.

[8] Thompson HJ, McGinley JN, Rothhammer K, Singh M (1995). Rapid induction of mammary intraductal proliferations, ductal carcinoma in situ and carcinomas by the injection of sexually immature female rats with 1-methyl-1-nitrosourea.. Carcinogenesis.

[9] Samuelson DJ, Hesselson SE, Aperavich BA, Zan Y, Haag JD (2007). Rat Mcs5a is a compound quantitative trait locus with orthologous human loci that associate with breast cancer risk.. Proc Natl Acad Sci U S A.

[10] Stingl J, Eirew P, Ricketson I, Shackleton M, Vaillant F (2006). Purification and unique properties of mammary epithelial stem cells.. Nature.

[11] Shackleton M, Vaillant F, Simpson KJ, Stingl J, Smyth GK (2006). Generation of a functional mammary gland from a single stem cell.. Nature.

[12] Visvader JE (2009). Keeping abreast of the mammary epithelial hierarchy and breast tumorigenesis.. Genes Dev.

[13] Dundas SR, Ormerod MG, Gusterson BA, O'Hare MJ (1991). Characterization of luminal and basal cells flow-sorted from the adult rat mammary parenchyma.. J Cell Sci.

[14] Asselin-Labat ML, Sutherland KD, Barker H, Thomas R, Shackleton M (2007). Gata-3 is an essential regulator of mammary-gland morphogenesis and luminal-cell differentiation.. Nat Cell Biol.

[15] Gugliotta P, Sapino A, Macri L, Skalli O, Gabbiani G (1988). Specific demonstration of myoepithelial cells by anti-alpha smooth muscle actin antibody.. J Histochem Cytochem.

[16] White DE, Kurpios NA, Zuo D, Hassell JA, Blaess S (2004). Targeted disruption of beta1-integrin in a transgenic mouse model of human breast cancer reveals an essential role in mammary tumor induction.. Cancer Cell.

[17] Li N, Zhang Y, Naylor MJ, Schatzmann F, Maurer F (2005). Beta1 integrins regulate mammary gland proliferation and maintain the integrity of mammary alveoli.. Embo J.

[18] Taddei I, Deugnier MA, Faraldo MM, Petit V, Bouvard D (2008). Beta1 integrin deletion from the basal compartment of the mammary epithelium affects stem cells.. Nat Cell Biol.

[19] Guan JL (2010). Integrin signaling through FAK in the regulation of mammary stem cells and breast cancer.. IUBMB Life.

[20] Luo M, Fan H, Nagy T, Wei H, Wang C (2009). Mammary epithelial-specific ablation of the focal adhesion kinase suppresses mammary tumorigenesis by affecting mammary cancer stem/progenitor cells.. Cancer Res.

[21] Zhao J, Guan JL (2009). Signal transduction by focal adhesion kinase in cancer.. Cancer Metastasis Rev.

[22] Kim ND, Clifton KH (1993). Characterization of rat mammary epithelial cell subpopulations by peanut lectin and anti-Thy-1.1 antibody and study of flow-sorted cells in vivo.. Exp Cell Res.

[23] Kornberg LJ, Earp HS, Turner CE, Prockop C, Juliano RL (1991). Signal transduction by integrins: increased protein tyrosine phosphorylation caused by clustering of beta 1 integrins.. Proc Natl Acad Sci U S A.

[24] Guan JL, Shalloway D (1992). Regulation of focal adhesion-associated protein tyrosine kinase by both cellular adhesion and oncogenic transformation.. Nature.

[25] Miller JA (1970). Carcinogenesis by chemicals: an overview--G. H. A. Clowes memorial lecture.. Cancer Res.

[26] Rudland PS, Barraclough R (1988). Stem cells in mammary gland differentiation and cancer.. J Cel lSci Supp.

[27] Rudland PS (1991). Generation of lobuloalveolar development from isolated rat mammary ducts and end buds.. J Histochem Cytochem.

[28] Rudland PS, Warburton MJ, Monaghan P, Ritter MA (1982). Thy-1 antigen on normal and neoplastic rat mammary tissues: changes in location and amount of antigen during differentiation of cultured stem cells.. J Natl Cancer Inst.

[29] Russo IH, Russo J (1978). Developmental stage of the rat mammary gland as determinant of its susceptibility to 7,12-dimethylbenz[a]anthracene.. J Natl Cancer Inst.

[30] Papaconstantinou AD, Shanmugam I, Shan L, Schroeder IS, Qiu C (2006). Gene expression profiling in the mammary gland of rats treated with 7,12-dimethylbenz[a]anthracene.. Int J Cancer.

[31] Ariazi JL, Haag JD, Lindstrom MJ, Gould MN (2005). Mammary glands of sexually immature rats are more susceptible than those of mature rats to the carcinogenic, lethal, and mutagenic effects of N-nitroso-N-methylurea.. Mol Carcinog.

[32] Shan L, Yu M, Snyderwine EG (2005). Gene expression profiling of chemically induced rat mammary gland cancer.. Carcinogenesis.

[33] Zarbl H, Sukumar S, Arthur AV, Martin-Zanca D, Barbacid M (1985). Direct mutagenesis of Ha-ras-1 oncogenes by N-nitroso-N-methylurea during initiation of mammary carcinogenesis in rats.. Nature.

[34] Lahlou H, Sanguin-Gendreau V, Zuo D, Cardiff RD, McLean GW (2007). Mammary epithelial-specific disruption of the focal adhesion kinase blocks mammary tumor progression.. Proc Natl Acad Sci U S A.

[35] Smits BM, Sharma D, Samuelson DJ, Woditschka S, Mau B (2011). The non-protein coding breast cancer susceptibility locus Mcs5a acts in a non-mammary cell-autonomous fashion through the immune system and modulates T-cell homeostasis and functions.. Breast Cancer Res.

[36] Clifton KH, Tanner MA, Gould MN (1986). Assessment of radiogenic cancer initiation frequency per clonogenic rat mammary cell in vivo.. Cancer Res.

